# Overcoming the Immunosuppressive Tumor Microenvironment in Multiple Myeloma

**DOI:** 10.3390/cancers13092018

**Published:** 2021-04-22

**Authors:** Fatih M. Uckun

**Affiliations:** 1 Norris Comprehensive Cancer Center and Childrens Center for Cancer and Blood Diseases, University of Southern California Keck School of Medicine (USC KSOM), Los Angeles, CA 90027, USA; fatih.uckun@aresmit.com; 2 Department of Developmental Therapeutics, Immunology, and Integrative Medicine, Drug Discovery Institute, Ares Pharmaceuticals, St. Paul, MN 55110, USA; 3 Reven Pharmaceuticals, Translational Oncology Program, Golden, CO 80401, USA

**Keywords:** tumor microenvironment (TME), multiple myeloma (MM), immunomodulatory imide drugs (IMiDs), autologous hematopoietic stem cell transplantation (ASCT), immune-checkpoint receptors, spleen tyrosine kinase (SYK), transforming growth factor beta (TGF-β), bispecific T-cell engagers (BiTEs), chimeric antigen receptor (CAR)-T

## Abstract

**Simple Summary:**

This article provides a comprehensive review of new and emerging treatment strategies against multiple myeloma that employ precision medicines and/or drugs capable of improving the ability of the immune system to prevent or slow down the progression of multiple myeloma. These rationally designed new treatment methods have the potential to change the therapeutic landscape in multiple myeloma and improve the long-term survival outcome.

**Abstract:**

SeverFigurel cellular elements of the bone marrow (BM) microenvironment in multiple myeloma (MM) patients contribute to the immune evasion, proliferation, and drug resistance of MM cells, including myeloid-derived suppressor cells (MDSCs), tumor-associated M2-like, “alternatively activated” macrophages, CD38+ regulatory B-cells (Bregs), and regulatory T-cells (Tregs). These immunosuppressive elements in bidirectional and multi-directional crosstalk with each other inhibit both memory and cytotoxic effector T-cell populations as well as natural killer (NK) cells. Immunomodulatory imide drugs (IMiDs), protease inhibitors (PI), monoclonal antibodies (MoAb), adoptive T-cell/NK cell therapy, and inhibitors of anti-apoptotic signaling pathways have emerged as promising therapeutic platforms that can be employed in various combinations as part of a rationally designed immunomodulatory strategy against an immunosuppressive tumor microenvironment (TME) in MM. These platforms provide the foundation for a new therapeutic paradigm for achieving improved survival of high-risk newly diagnosed as well as relapsed/refractory MM patients. Here we review the scientific rationale and clinical proof of concept for each of these platforms.

## 1. Introduction

**Figure 1:**
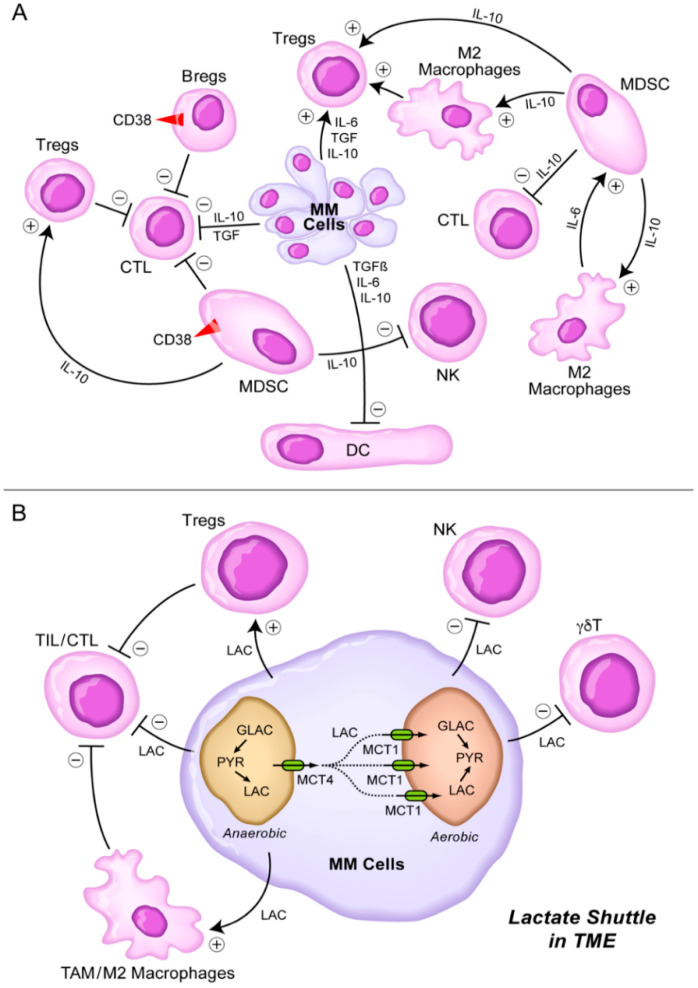
Immunosuppressive TME in MM. (**A**) MM cells secrete several cytokines including IL-6, TGF-β (TGF), and IL-10 [7,8,9,10] that inhibit DCs, CTLs, but stimulate Tregs. MDSCs are stimulated by M2 macrophages via IL-6 and stimulate M2 macrophages as well as Tregs via IL-10. MDSC inhibit CTLs and NK cells via IL-10. See text for detailed discussion. (**B**) Lactate shuttle in TME. Lactate derived from MM cells stimulates Tregs and M2 macrophages, but it inhibits TILs, CTL, NK cells, and γδT cells. See text for detailed discussion.

The tumor microenvironment (TME) is one of the main contributors of a marked immunobiological and clinical heterogeneity as well as clonal evolution in multiple myeloma (MM) [1,2,3]. Several cellular elements of the bone marrow (BM) microenvironment in MM patients contribute to the immune evasion, proliferation, and drug resistance of MM cells [4,5,6], including myeloid-derived suppressor cells (MDSCs), tumor-associated M2-like, “alternatively activated”, macrophages, CD38^+^ regulatory B-cells (Bregs), and regulatory T-cells (Tregs) [4,5,6] (Figure 1A). These immunosuppressive elements in bidirectional and multi-directional crosstalk with each other inhibit both memory and cytotoxic effector T-cell populations as well as natural killer (NK) cells [4,5,6]. MDSCs along with MM cell-derived interleukin 10 (IL-10) [7,8,9,10], TGF-β, and IL-6 are also known to impair dendritic cell (DC) maturation and their antigen-presenting function, which further accentuates the immunosuppression [6]. In addition to their immune-suppressive activity mediated by their secretion of IL-10 (an activator of Tregs and M2 macrophages), and TGF-β (an inhibitor of both cytotoxic T-cells and NK cells), M2 macrophages also promote MM cell proliferation, angiogenesis, and chemotherapy resistance [11,12,13] (Figure 1A). Nonclinical studies in animal models of MM have demonstrated that exosomes serve as regulators of the signaling networks within the BM microenvironment, activate anti-apoptotic mechanisms promoted by oncogenic proteins such as the signal transducer and activator of transcription 3 (STAT3), and cause immunosuppression by facilitating the growth of MDSCs [14,15,16,17,18,19]. Exosome-activated MDSCs have been implicated in development of other immunosuppressive cells, such as Tregs, tumor-promoting angiogenesis, proliferation of MM cells, and increased osteoclast activity contributing to the lytic bone lesions [14,15,16,17,18,19]. Complementing the immunosuppressive TME, T-cell exhaustion combined with high-level expression of immune-checkpoint ligands on MM cells are the main contributors to the immune evasion of MM cells [20,21,22,23]. 

## 2. Immunomodulatory Imide Drugs (IMiDs) to Overcome the Immunosuppressive TME in MM

IMiDs augment the activity of cereblon E3 ubiquitin ligase complex and enhance the effector functions as well as the proliferative expansion of cytotoxic T-cells and NK cells through cereblon-dependent proteolytic degradation of the transcription factors Ikaros (IKZF1) and Aiolos (IKZF3), increased production of IL-2 and IFN-γ with concomitant reduction in IL-10 production and suppression of Tregs [24,25,26,27]. They also block the pro-inflammatory cytokines TNF-α and IL-6, thereby decreasing the contributions of MDSC and M2 macrophages to the immunosuppressive milieu of the TME. Notably, in a IMiD-conditioned TME, T-cell activation is facilitated, as it no longer requires co-stimulation by antigen-presenting DCs [24,25,26,27]. In addition, IMiDs trigger caspase-mediated apoptosis in MM cells and impair their interaction with the protective BM stroma [28]. Efforts are underway to further enhance the clinical potency of IMiDs by combining them with modulators of cereblon E3-ligase, such as iberdomide, that accelerate IMiD-mediated degradation of IKZF1/IKZF3 [26,27]. Due to the considerable added benefit of the IMiD thalidomide, a combination of bortezomib, thalidomide, and dexamethasone (VTD) has become a standard induction regimen [29]. New generation IMiDs, including lenalidomide (Revlimid; Celgene) and pomalidomide (Pomalyst; Celgene) have potent immune modulatory effects and have significantly improved the survival outcomes of newly diagnosed as well as R/R MM patients [30]. IMiDs are used in combination with Proteasome inhibitors (PIs), such as bortezomib, carfilzomib, or ixazomib, in standard of care for MM to achieve optimal anti-tumor activity as well as inhibit angiogenesis within the TME [30]. Recent studies indicated that PIs also affect the TME in MM patients by disrupting MM–stroma interactions, angiogenesis, and bone remodeling [31]. In addition, they are believed to contribute to sustained immune responses by inducing an immunogenic death [32]. 

## 3. Autologous Hematopoietic Stem Cell Transplantation as an Immunomodulatory Strategy against Immunosuppressive TME in MM

Autologous hematopoietic stem cell transplantation (ASCT) has also been proposed as an evolving strategy to overcome the immunosuppressive TME in MM, and the focal point of contemporary research in this context is the determination of how best to integrate ASCT into standard of care in the context of precision medicines [30,32,33]. It has been proposed that an immunosuppressive BM microenvironment in MM can be remodeled by myeloablative and lymphodepleting pretransplant conditioning in the context of ASCT to re-establish a favorable effector T-cell/Treg ratio with effective immune surveillance mechanisms by MM-specific memory and effector T-cells that can prevent disease progression [23]. Additional treatment strategies aimed at further enhancing the anti-MM immunity, such as immune-checkpoint inhibitors (ICIs), bispecific T-cell engagers (BiTEs), and CAR-T cells, can be employed post-ABMT [23]. Therefore, using ASCT as a salvage platform in patients with R/R MM, new multimodality strategies, including biotherapeutic agents such as BiTEs and CAR-T cell platforms, are being evaluated as post-ASCT interventions [33]. 

## 4. Rationale of Targeting CD38, SLAMF7, CD137, and KIR as an Immunomodulatory Strategy against Immunosuppressive TME in MM

**Figure 2:**
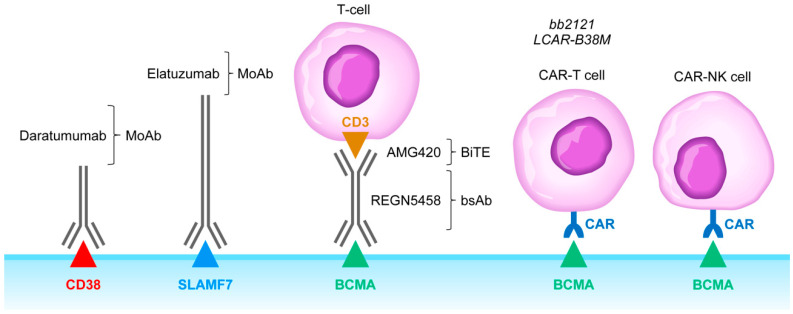
Monoclonal antibodies, BiTEs, BsAbs, CAR-T cells, and CAR-NK cells targeting MM cells. Abbreviations: MoAb: monoclonal antibody; BiTE: bispecific T-cell engager; bsAb: bispecific antibody; CAR-T: chimeric antigen receptor carrying T-cell; CAR-NK: chimeric antigen receptor carrying T-cell. See text for a detailed discussion of the rationale of targeting the CD38, SLAMF7, and BCMA receptors.

CD38 surface antigen is abundantly expressed on MM cells [30,34,35]. Anti-CD38 MoAb, including daratumumab (DARA), isatuximab (ISA), and MOR202 (Figure 2), have shown tolerability as well as meaningful clinical activity in FDA-approved monotherapy regimens and as components of FDA-approved multi-modality combination regimens for treatment of R/R MM patients [30,34,35,36]. Recently, a new formulation of DARA containing recombinant human hyaluronidase PH20 has become available that can be administered subcutaneously [37]. DARA has increased the efficacy of the standard VLD regimen in ASCT-eligible patients and contributed to deep CRs with MRD negativity in the randomized GRIFFIN trial [38]. 

CD38-targeting MoAb such as DARA have been shown to cause complement-dependent cytotoxicity (CDC), antibody-dependent cellular cytotoxicity (ADCC), antibody-dependent cellular phagocytosis (ADCP), and tumor cell apoptosis [30]. However, CD38 is expressed on immunosuppressive cellular elements of the TME as well, including MDSCs and regulatory B-cells (Bregs) [2,20,23,30]. Krejcik et al. reported that DARA is capable of depleting CD38^+^ immune regulatory cells, thereby increasing the size of the immunoreactive clonal cytotoxic effector T-cell populations [39]. Biomarker analyses in R/R MM patients using a next-generation mass cytometry platform suggested a novel immunomodulatory mechanism of action associated with the activation of CD8^+^ cytotoxic T-cells [40]. The immunomodulatory effects of DARA and other anti-CD38 MoAb may be useful to achieve clinical responses in IMiD-refractory MM patients using IMiD in salvage therapy following DARA monotherapy [30]. 

The CD38 antibodies also improve host-anti-tumor immunity by elimination of regulatory T-cells, regulatory B-cells, and MDSCs [41,42]. Mechanisms of primary and/or acquired resistance include tumor-related factors. It has been shown that increased expression levels of complement inhibitors CD55 and CD59 as well as decreased cell surface expression levels of CD38 on MM cells may confer resistance to anti-CD38 MoAb [35], whereas certain KIR and HLA genotypes are associated with higher effectiveness of treatment regimens employing anti-CD38 MoAb [35]. Therefore, biomarker-guided patient-tailored application of anti-CD38 MoAb may further improve their clinical impact potential in MM therapy.

SLAMF7 surface antigen is expressed on both MM cells and plays an important role in MM cell adhesion to protective BM stroma (Figure 2). SLAMF7 has been implicated in negative regulation of NK cell function as well. The FDA-approved anti-SLAMF7 MoAb elotuzumab impairs the viability and survival of MM cells by blocking the protective effects of stromal cells and by potentiating NK cell activity against MM cells [30,43]. A Phase 3 clinical trial in R/R MM (ELOQUENT-2, ClinicalTrials.gov identifier, NCT01239797) demonstrated the addition of elotuzumab to lenalidomide and dexamethasone significantly improves the survival outcomes when compared to lenalidomide plus dexamethasone combination [30,44]. Likewise, the addition of elotuzumab topomalidomide plus dexamethasone resulted in significantly better survival outcomes in another Phase 3 study (ELOQUENT-3, ClinicalTrials.gov identifier: NCT02654132) [45]. Unfortunately, inclusion of this immunostimulatory antibody in combination regimens against MM resulted in a higher incidence of infections and secondary cancers [46]. Other immunostimulatory antibodies have been considered in combination regimens with elotuzumab against MM, including the T-cell activating 4-1BB/CD137 agonist antibodies urelumab or utomilumab and the NK cell activating anti-KIR antibody lirilumab (NCT02252263) [47,48]. Due to potentially life-threatening hepatotoxicity of urelumab, some of these combination regimens will require specifically designed risk mitigation strategies. 

## 5. Targeting Immune-Checkpoint Receptors in MM

As T-cell exhaustion combined with high-level expression of immune-checkpoint receptors on MM cells have been implicated in the immune evasion of MM cells, ICIs targeting the PD-1/PD-L1 axis have been studied as components of novel multi-modality regimens in R/R MM. Unfortunately, the more widespread clinical use of these promising immune-oncology drugs has been hampered by a lack of single agent activity in monotherapy studies and by serious safety concerns and increased mortality especially when they are combined with IMiDs [21,30,49,50]. In Keynote 183 study, a combination of pembrolizumab, lenalidomide, and dexamethasone was used for treatment of newly diagnosed MM patients, whereas in the Keynote 185 study, a similar combination, namely, pembrolizumab, pomalidomide, and dexamethasone was used in R/R MM patients [51]. The addition of pembrolizumab was associated with more deaths but not with greater efficacy, and both studies were terminated [51]. While the reasons for the failure of immune-checkpoint plus IMiD combination therapy in MM remain to be deciphered, it has been proposed that the observed toxicities and organ damage may be due to activation of virus-specific memory cells [51]. 

Poor innate immunity owing to NK cell impairment in MM also contributes to the immune escape of MM cells. Therefore, restoration of effective NK cell function would be highly desirable. The lymphocyte activation gene-3 (LAG3) inhibitory receptor (CD223) is an immune-checkpoint receptor widely expressed on not only T-cells but also NK cells. Anti-LAG3 antibodies such as relatlimab are capable of triggering activation of both cytotoxic T-cells and NK cells in the MM TME [22,52,53]. Other antibodies targeting alternative inhibitory NK cell receptors, including KIRs, NKG2A, TIGIT, and TIM-3, are also being explored (Clinicaltrials.gov identifier: NCT04150965).

## 6. Clinical Impact Potential of Bispecific T-Cell Engagers and Adoptive T-Cell/NK Cell Therapy Against the Immunosuppressive TME in MM

Bispecific T-cell engagers (BiTEs) developed for oncologic indications contain a T-cell engaging moiety that binds to a T-cell surface antigen (typically CD3) and a tumor-associated antigen that is expressed on malignant cells. BiTEs and bispecific antibodies bring T-cells and targeted tumor cells in close vicinity of each other in cytolytic synapses, which leads to tumor-specific cytolytic T-cell activation and MM cell lysis [30,54,55,56,57,58]. BiTEs targeting the MM-associated B-cell maturation antigen (BCMA) have been associated with very promising, albeit short-lived, efficacy signals raising hopes that they may provide the foundation for effective strategies against the immune-suppressive TME in MM [30,54,55,56,57,58] (Figure 2). Bispecific BCMAxCD3 antibodies such as Regeneron’s REGN5458 also showed clinical potential with promising data from recent clinical trials in R/R MM patients [54] (Figure 2). In view of the lessons and insights learned from the clinical development and related clinical trials of BLINCYTO, clinical protocols employing BiTEs or bsAbs targeting MM cells should consider incorporating monitoring and management guidelines for risk mitigation regarding neurologic toxicities (e.g., encephalopathy, convulsions, speech disorders, disturbances in consciousness, confusion and disorientation, and coordination and balance disorders), disseminated intravascular coagulation (DIC), capillary leak syndrome (CLS)/cytokine release syndrome (CRS), pancreatitis, leukoencephalopathy and hemophagocytic lymphohistiocytosis/macrophage activation syndrome (HLH/MAS). 

Likewise, BCMA-specific chimeric antigen receptor (CAR)-T platforms and CAR-NK cells have been developed for adoptive cell therapy in MM [55,59,60,61,62,63,64] (Figure 2). Some of the CAR-T platforms induced complete remissions (CRs) in R/R MM patients that were durable [30,60,65,66]. Similar to BiTEs, CAR-T platforms are associated with potentially life-threatening side effects, such as CRS, neurotoxicity, and severe immune-related AEs, which necessitates specific risk mitigation strategies aimed at early detection and immediate treatment of the complications [30]. Specific algorithms that have been developed for management of CLS/CRS in leukemia patients, including the use of anti-TNF receptor (e.g., infliximab) or anti-IL-6 receptor (e.g., tocilizumab) treatments in cases not responsive to steroids, are likely to be helpful in managing MM patients who experience these complications because of their treatment with BCMA-targeting BiTES/bsAbs or CAR-T cells. A clinical proof-of-concept study (Clinicaltrials.gov identifier: NCT0302577) is underway to reduce the levels of γ-secretase cleaved soluble BCMA in MM patients by using a γ-secretase inhibitor, because soluble BCMA interferes with the efficacy of both BCMA-directed BiTEs and CAR-T cells [65]. Early clinical data regarding the use of CAR-T cells post ASCT are promising [22,66]. The orphan G protein-coupled receptor, class C group 5 member D (*GPRC5D*) protein is abundantly expressed on MM cells, and its expression profile is similar to that of the BCMA antigen [67]. Therefore, GPRC5D protein, similar to BCMA, represents an attractive target for monoclonal antibody-based (e.g., bispecific T-cell-engaging antibodies or antibody–drug conjugates) or cell-based (e.g., CAR-T or CAR-NK) immunotherapy in MM. 

## 7. Targeting Transforming Growth Factor Beta (TGF-β) Signaling and SYK-PI3K-AKT Pathway

TGF-β can limit the T-cell infiltration to the TME and inhibiting T-cells as well as NK-cells, which leads to a markedly diminished anti-tumor immune response within the TME. TGF-β has been identified as a major cause of T-cell exhaustion and hyporesponsiveness in the context of an immunosuppressive TME [68,69,70,71,72,73]. TGF-β is also a contributing cause of the T-cell exclusion from TME as well as failure of immune-oncology drugs targeting the immune-checkpoint receptors to boost the host anti-tumor immunity [71,72,73,74]. Targeting the TGF-β pathway has been associated with durable CRs and PRs in difficult to treat cancers, such as high-grade gliomas and pancreas cancer [70]. Combining immune-checkpoint inhibitors with inhibitors of the TGF-β pathway has induced meaningful clinical responses in PD-1/PD-L1-resistant cancers [72,73,74]. 

Interleukin-18 (IL-18), the signature pro-inflammatory cytokine of the TGF-β signaling pathway, serves as an immunosuppressive regulator within the TME of MM patients by supporting the growth of MDSCs that block T-cell immune responses against MM cells and thereby contributes to disease progression [13]. Nakamura et al. reported that high levels of IL-18 in the bone marrow is a predictor of poor survival outcome in MM [13]. Notably, MM cells have upregulated expression of TGF-β-activated kinase-1 (TAK1), and inhibiting TAK1 has been shown to significantly impair the growth and survival of MM cells [75]. Therefore, targeting TGF-β signaling with an RNA therapeutic such as OT101, the TGF-β targeting anti-inflammatory platforms such as RJX [76] or ArtiVeda [77,78] or a small molecule inhibitor such as galunisertib could contribute to sustained anti-tumor immunity and PD-1/PD-L1 responsiveness by lifting TGF-β-mediated immunosuppression. 

**Figure 3:**
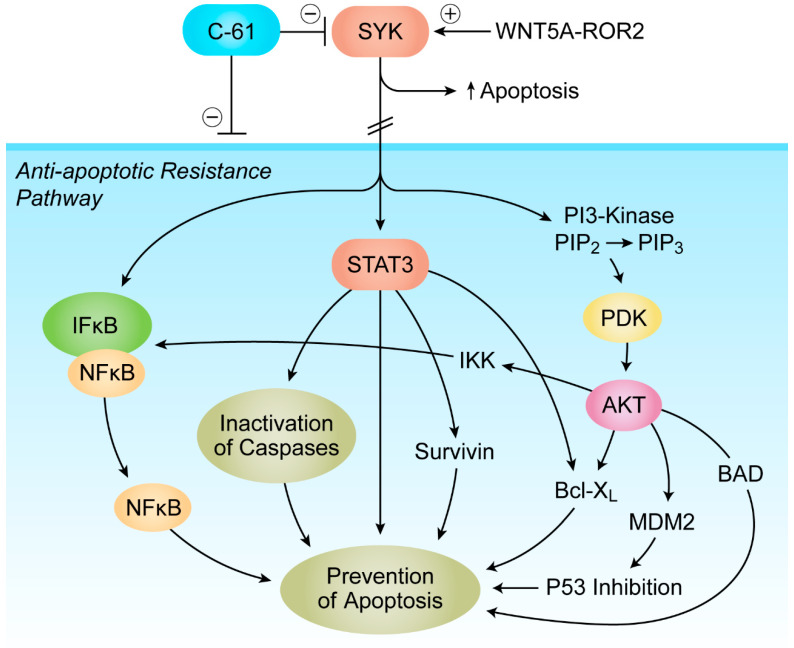
SYK as a master regulator of apoptosis in MM TME. The receptor tyrosine kinase ROR2 is the receptor for WNT5A, a growth factor that is abundantly expressed in the TME of MM patients. WNT5A–ROR2 interactions within the TME promote the adhesion of MM cells to the bone marrow niche and activate the anti-apoptotic SYK-PI3K-AKT signaling pathway, which is critical for the survival of MM cells [82]. Multiple anti-apoptotic signaling molecules and pathways linked to NFκB, PI3-K/AKT, and STAT3 are regulated by SYK. See text for detailed discussion.

**Figure 4:**
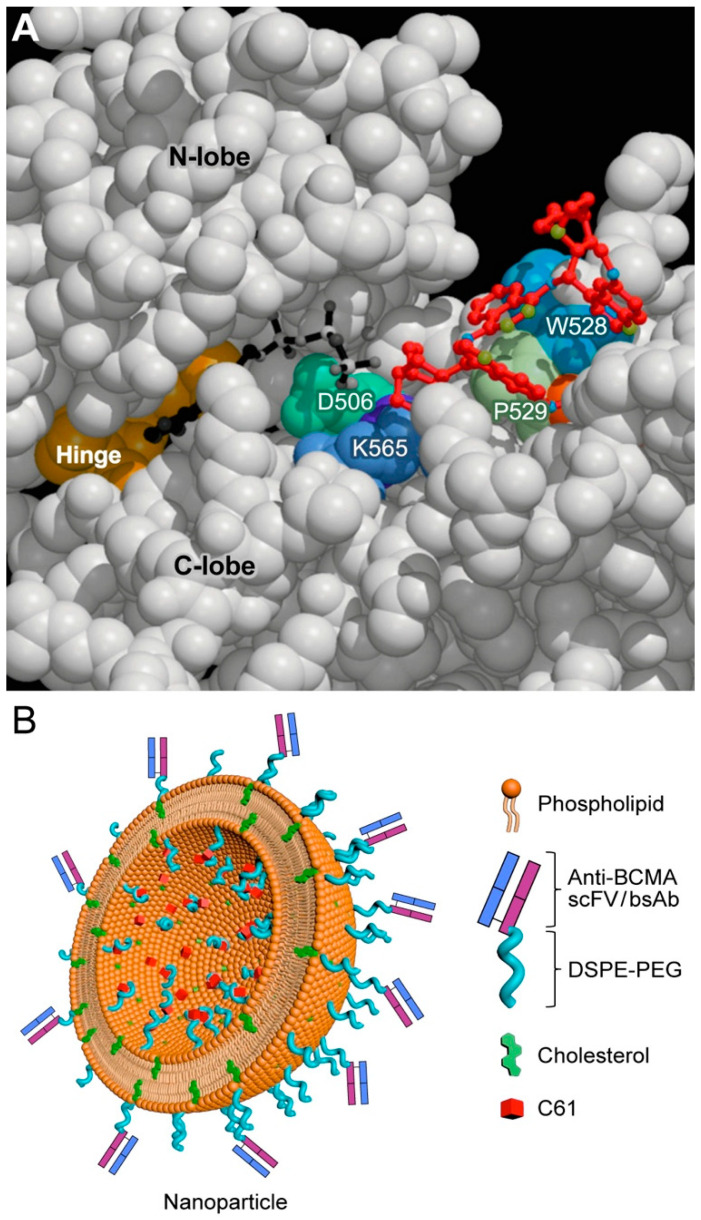
Nanoparticles targeting SYK pathway in MM cells. (**A**) Model of the SYK inhibitor C61 (1,4-Bis (9-O-dihydroquinidinyl) phthalazine/hydroquinidine 1,4-phathalazinediyl diether) molecule (multicolor) bound to the protein substrate-binding site of SYK kinase domain in the central region of SYK, between the N-lobe and C-lobe, which includes multiple residues such as K565, P529, and W528. Nearby ATP molecule: black and white. (**B**) Cartoon illustration of a BCMA-directed PEGylated liposomal nanoparticle formulation of the SYK P-site inhibitor C61. Such nanoparticles can be prepared by using the thin film evaporation method with the use of dipalmitoyl phosphatidylcholine (DPPC), cholesterol, 1,2-distearoyl-sn-glycero-3-phosphoethanolamine-*N*-[methoxy(polyethylene glycol)-2000] (DSPE-PEG2000), and the entrapment of C61 within the interior space of LNP can be achieved using a pH gradient procedure that employs lactobionic acid (LBA), as described [85].

Other cytokines implicated in the pathobiology of MM include CSF-1, IL-6, IL-17, IL-21, IL-23, VEGF, growth differentiating factor 15, IGF-1, and myeloma-cell-secreted type 1 IFN [5,11,79,80,81]. CSF1 is the main driver of differentiation of M1 macrophages into immunosuppressive M2 macrophages. As such, CSF1 plays an important role in progression of disease in MM [79]. IL-6 is a major growth and chemotherapy resistance factor for MM cells that triggers signaling pathways contributing to proliferation and anti-apoptotic resistance [5,11]. The receptor tyrosine kinase ROR2 is the receptor for WNT5A, a growth factor that is abundantly expressed in the TME of MM patients. WNT5A–ROR2 interactions within the TME promote the adhesion of MM cells to the bone marrow niche and activate the anti-apoptotic SYK-PI3K-AKT signaling pathway, which is critical for the survival of MM cells [82] (Figure 3). Targeting SYK with small molecule kinase inhibitors has been shown to cause apoptotic death and growth arrest of MM cells [83,84]. Therefore, new-generation nanoscale SYK inhibitors capable of better TME infiltration [85] (Figure 4) show clinical impact potential not only because of their cytotoxic activity but also because of their potential to abrogate the WNT5A-mediated MM adhesion to the protective stroma of the TME. 

Another potentially attractive strategy for overcoming an immunosuppressive TME in MM would be the application of various platforms, such as agonistic CD40 monoclonal antibodies, capable of molecular repolarization of tumor-associated macrophages with a proinflammatory phenotype, especially if the off-target risk of systemic inflammation can be mitigated [86,87].

Several tumor vaccines against MM-associated antigens, including MAGA-C1, MUC-1, WT-1, NY-ESO-1 as well as idiotypic IgG, have been developed and combined with strategies such as dendritic cell (DC) vaccination, to be employed for immune vaccination in high-risk MM patients either alone or in combination with immune-checkpoint inhibitors [88,89]. It has also been reported that oncolytic measles virus therapy can be applied to improve host T-cell immunity against MM by causing immunogenic death of virus-infected MM cells [90]. 

## 8. Targeting the Lactate Shuttle in the TME

Lactate is both a product and an energy source of cellular respiration. After glucose yields pyruvic acid from glycolysis, pyruvic acid is processed differently based on how much oxygen the cell has available. In an aerobic environment, pyruvic acid produces energy in the tricarboxylic acid (i.e., citric acid) cycle, and no lactate is produced. In an anaerobic environment, pyruvic acid is reduced by the enzyme lactate dehydrogenase (LDH) to lactate. Cancer cells, including MM cells, often shift the focus of their metabolic processes toward anaerobic cycles that produce lactate [91,92,93,94,95,96]. Lactate has even been shown to be preferred over glucose as an energy source by cancer cells in a well-oxygenated environment [91,92,93,94]. Usually, this would make the tumor cells less energetically efficient by adding more steps to the cellular respiration process. However, it is now known that optimization to secrete and consume lactate leads to the emergence of a regionally efficient metabolism for the tumor taken as a whole. MM cells are not always close enough to blood vessels to be well oxygenated. Anaerobic processes make sense for the cells in these areas, and so they produce significant amounts of lactate. If these cells are examined by themselves, then their lactate production seems wasteful and likely to contribute to detrimental amounts of lactic acid in their local environment. However, the excess lactate they secrete is taken up via monocarboxylate transporters (MCT) by other MM cells in the same region that have better access to vasculature. In the better-oxygenated cells, lactate dehydrogenase (LDH) converts the lactate back to pyruvic acid and uses it for energy. This lactate shuttle pathway from oxygen-poor regions to oxygen-rich regions provides the oxygenated MM cells with an abundance of energy from lactate (Figure 1B). MM cell-derived lactate promotes the development of immunosuppressive Tregs and MDSCs [91]. Macrophages can sense lactate secreted from tumor cells and respond with immunosuppressive activity [92,93,94]. Lactate inhibits NK cells, gdT cells, TILs, and CTLs (Figure 1B). Both lactate and LDH in the tumor microenvironment can facilitate the protumor activity of tumor-associated macrophages [92,93,94].

MM cells specifically use lactate to mediate several environment changes that support tumor growth and stimulate more lactate production. Significant accumulations of lactate activate transcription factor hypoxia-inducible factor-1 *α* (HIF-1*α*) in MM cells [95,96]. This activation occurs when lactate is taken into a cell by monocarboxylate transporter 1 (MCT1). HIF-1*α* is responsible for activating and mediating many of the responses of a cell to an anaerobic environment [96]. Furthermore, HIF-1α is constitutively active in more than a third of patients with MM [95]. HIF-1*α* increases rates of glycolysis, increases MCT activation, suppresses immune reactions from T-cells, and stimulates release of vascular endothelial growth factor (VEGF). Increased rates of glycolysis increase energy and carbon availability. MCT activation transports more lactate into and out of cells. T-cell suppression increases the tumor’s evasion of immune responses and immunotherapy. VEGF stimulates new blood vessels to form, supplying more oxygen and nutrients to the cell. Sufficient lactate accumulation to activate HIF-1*α* relies typically on an absence of oxygen to increase lactate production from pyruvic acid. However, MM cells can also use an intercellular “lactate shuttle” to create an oxygen-independent pathway to this accumulation. The activation of HIF-1 *α* increases lactate production by increased rates of glycolysis. A cyclical signaling pathway forms within the TME, whereby cells produce and secrete large amounts of lactate, which induce neighboring cells to produce and secrete more lactate as well. The tumor maintains its lactate energy surplus in this way and gains the additional benefits of increased vascular growth into the tumor and increased immunosuppression around it.

Targeting the “lactate shuttle” by facilitating lactate clearance may eliminate the contribution of lactate to the immunosuppressive TME in MM. It is noteworthy that thiamine and magnesium sulphate that are ingredients of the anti-inflammatory, anti-oxidant investigational drug product RJX [81], which is being developed as a treatment platform against CRS, have been shown to facilitate lactate clearance in patients with sepsis. Likewise, targeting metabolic pathways that play a role in the development of a hypoxic TME in MM may provide the basis for innovative strategies with clinical impact potential [91]. 

## 9. Future Directions

New drugs and strategies capable of overcoming the immunosuppressive TME would have very high clinical impact potential for more effective treatment of high-risk and R/R MM. The identification of the most effective and best tolerated combination regimens will require rationally designed clinical studies with multiple treatment cohorts enrolling in parallel and adaptive trial designs. 

## 10. Conclusions

The therapeutic landscape for MM has been rapidly evolving due to introduction of targeted precision medicines, IMiDs, monoclonal antibodies, BiTEs, and CAR-T cells into multi-modality treatment strategies that have improved the survival outcomes [97,98,99,100,101,102,103,104,105,106,107,108,109].

## References

[1] Schürch C.M., Rasche L., Frauenfeld L., Weinhold N., Fend F. (2019). A review on tumor heterogeneity and evolution in multiple myeloma: Pathological, radiological, molecular genetics, and clinical integration. Virchows Arch..

[2] Holthof L.C., Mutis T. (2020). Challenges for Immunotherapy in Multiple Myeloma: Bone Marrow Microenvironment-Mediated Immune Suppression and Immune Resistance. Cancers.

[3] Ribatti D., Nico B., Vacca A. (2006). Importance of the bone marrow microenvironment in inducing the angiogenic response in multiple myeloma. Oncogene.

[4] Kawano Y., Moschetta M., Manier S., Glavey S., Görgün G.T., Roccaro A.M., Anderson K.C., Ghobrial I.M. (2015). Targeting the bone marrow microenvironment in multiple myeloma. Immunol. Rev..

[5] García-Ortiz A., Rodríguez-García Y., Encinas J., Maroto-Martín E., Castellano E., Teixidó J., Martínez-López J. (2021). The Role of Tumor Microenvironment in Multiple Myeloma Development and Progression. Cancers.

[6] Caraccio C., Krishna S., Phillips D.J., Schürch C.M. (2020). Bispecific Antibodies for Multiple Myeloma: A Review of Targets, Drugs, Clinical Trials, and Future Directions. Front Immunol..

[7] Kovacs E. (2010). Interleukin-6 leads to interleukin-10 production in several human multiple myeloma cell lines. Does interleukin-10 enhance the proliferation of these cells?. Leuk. Res..

[8] Otsuki T., Yamada O., Yata K., Sakaguchi H., Kurebayashi J., Yawata Y., Ueki A. (2000). Expression and production of interleukin 10 in human myeloma cell lines. Br. J. Haematol..

[9] Otsumi T., Yata K., Sakaguchi H., Uno M., Fujii T., Wada H., Sugihara T., Ueki A. (2002). IL-10 in Myeloma Cells. Leuk. Lymphoma.

[10] Matsumoto M., Baba A., Yokota T., Nishikawa H., Ohkawa Y., Kayama H., Kallies A., Nutt S.L., Sakaguchi S., Takeda K. (2014). Interleukin-10-Producing Plasmablasts Exert Regulatory Function in Autoimmune Inflammation. Immunity.

[11] Ostrand-Rosenberg S., Sinha P., Beury D.W., Clements V.K. (2012). Cross-talk between myeloid-derived suppressor cells (MDSC), macrophages, and dendritic cells enhances tumor-induced immune suppression. Semin. Cancer Biol..

[12] Urashima M., Ogata A., Chauhan D., Hatziyanni M., Vidriales M., Dedera D., Schlossman R., Anderson K. (1996). Transforming growth factor-beta1: Differential effects on multiple myeloma versus normal B cells. Blood.

[13] Nakamura K., Kassem S., Cleynen A., Chrétien M.-L., Guillerey C., Putz E.M., Bald T., Förster I., Vuckovic S., Hill G.R. (2018). Dysregulated IL-18 Is a Key Driver of Immunosuppression and a Possible Therapeutic Target in the Multiple Myeloma Microenvironment. Cancer Cell.

[14] Chen T., Moscvin M., Bianchi G. (2020). Exosomes in the Pathogenesis and Treatment of Multiple Myeloma in the Context of the Bone Marrow Microenvironment. Front. Oncol..

[15] Moloudizargari M., Abdollahi M., Asghari M.H., Zimta A.A., Neagoe I.B., Nabavi S.M. (2019). The emerging role of exosomes in multiple myeloma. Blood Rev..

[16] Li M., Xia B., Wang Y., You M.J., Zhang Y. (2019). Potential Therapeutic Roles of Exosomes in Multiple Myeloma: A Systematic Review. J. Cancer.

[17] Raimondo S., Urzì O., Conigliaro A., Bosco G.L., Parisi S., Carlisi M., Siragusa S., Raimondi L., de Luca A., Giavaresi G. (2020). Extracellular Vesicle microRNAs Contribute to the Osteogenic Inhibition of Mesenchymal Stem Cells in Multiple Myeloma. Cancers.

[18] Raimondi L., de Luca A., Fontana S., Amodio N., Costa V., Carina V., Bellavia D., Raimondo S., Siragusa S., Monteleone F. (2020). Multiple Myeloma-Derived Extracellular Vesicles Induce Osteoclastogenesis through the Activation of the XBP1/IRE1α Axis. Cancers.

[19] Wang J., de Veirman K., Faict S., Frassanito M.A., Ribatti D., Vacca A., Menu E. (2016). Multiple myeloma exosomes establish a favourable bone marrow microenvironment with enhanced angiogenesis and immunosuppression. J. Pathol..

[20] Soekojo C.Y., Ooi M., De Mel S., Chng W.J. (2020). Immunotherapy in Multiple Myeloma. Cells.

[21] Costello C. (2019). The future of checkpoint inhibition in multiple myeloma?. Lancet Haematol..

[22] Yamamoto L., Amodio N., Gulla A., Anderson K.C. (2021). Harnessing the Immune System Against Multiple Myeloma: Challenges and Opportunities. Front. Oncol..

[23] Minnie S.A., Hill G.R. (2020). Immunotherapy of multiple myeloma. J. Clin. Investig..

[24] Krönke J., Udeshi N.D., Narla A., Grauman P., Hurst S.N., McConkey M., Svinkina T., Heckl D., Comer E., Li X. (2014). Lenalidomide causes selective degradation of IKZF1 and IKZF3 in multiple myeloma cells. Science.

[25] Lu G., Middleton R.E., Sun H., Naniong M., Ott C.J., Mitsiades C.S., Wong K.K., Bradner J.E., Kaelin W.G. (2014). The myeloma drug lenalidomide promotes the cereblon-dependent destruction of Ikaros proteins. Science.

[26] Bjorklund C.C., Kang J., Amatangelo M., Polonskaia A., Katz M., Chiu H., Couto S., Wang M., Ren Y., Ortiz M. (2020). Iberdomide (CC-220) is a potent cereblon E3 ligase modulator with antitumor and immunostimulatory activities in lenalidomide- and pomalidomide-resistant multiple myeloma cells with dysregulated CRBN. Leukemia.

[27] Hansen J.D., Correa M., Nagy M.A., Alexander M., Plantevin V., Grant V., Whitefield B., Huang D., Kercher T., Harris R. (2020). Discovery of CRBN E3 Ligase Modulator CC-92480 for the Treatment of Relapsed and Refractory Multiple Myeloma. J. Med. Chem..

[28] Gulla A., Anderson K.C. (2020). Multiple myeloma: The (r)evolution of current therapy and a glance into future. Haematology.

[29] Lee J.H., Kim S.-H. (2020). Treatment of relapsed and refractory multiple myeloma. Blood Res..

[30] Uckun F.M., Qazi S., Demirer T., Champlin R.E. (2019). Contemporary patient-tailored treatment strategies against high risk and relapsed or refractory multiple myeloma. EBioMedicine.

[31] Gandolfi S., Laubach J.P., Hideshima T., Chauhan D., Anderson K.C., Richardson P.G. (2017). The proteasome and proteasome inhibitors in multiple myeloma. Cancer Metastasis Rev..

[32] Valle A.S.-D., Anel A., Naval J., Marzo I. (2019). Immunogenic Cell Death and Immunotherapy of Multiple Myeloma. Front. Cell Dev. Biol..

[33] Al Hamed R., Bazarbachi A.H., Malard F., Harousseau J.-L., Mohty M. (2019). Current status of autologous stem cell transplantation for multiple myeloma. Blood Cancer J..

[34] Van de Donk N.W.C.J., Richardson P.G., Malavasi F. (2018). CD38 antibodies in multiple myeloma: Back to the future. Blood.

[35] Chong L.L., Soon Y.Y., Soekojo C.Y., Ooi M., Chng W.J., de Mel S. (2021). Daratumumab-based induction therapy for multiple myeloma: A systematic review and meta-analysis. Crit. Rev. Oncol. Hematol..

[36] Giri S., Grimshaw A., Bal S., Godby K., Kharel P., Djulbegovic B., Dimopoulos M.A., Facon T., Usmani S.Z., Mateos M.-V. (2020). Evaluation of Daratumumab for the Treatment of Multiple Myeloma in Patients with High-risk Cytogenetic Factors. JAMA Oncol..

[37] Mateos M.-V., Nahi H., Legiec W., Grosicki S., Vorobyev V., Spicka I., Hungria V., Korenkova S., Bahlis N., Flogegard M. (2020). Subcutaneous versus intravenous daratumumab in patients with relapsed or refractory multiple myeloma (COLUMBA): A multicentre, open-label, non-inferiority, randomised, phase 3 trial. Lancet Haematol..

[38] Voorhees P.M., Kaufman J.L., Laubach J.P., Sborov D.W., Reeves B., Rodriguez C., Chari A., Silbermann R., Costa L.J., Anderson L.D. (2020). Daratumumab, lenalidomide, bortezomib, and dexamethasone for transplant-eligible newly diagnosed multiple myeloma: The GRIFFIN trial. Blood.

[39] Krejcik J., Casneuf T., Nijhof I.S., Verbist B., Bald J., Plesner T., Syed K., Liu K., van de Donk N.W., Weiss B.M. (2016). Daratumumab depletes CD38+ immune regulatory cells, promotes T-cell expansion, and skews T-cell repertoire in multiple myeloma. Blood.

[40] Adams H.C., Stevenaert F., Krejcik J., van der Borght K., Smets T., Bald J., Abraham Y., Ceulemans H., Chiu C., Vanhoof G. (2019). High-Parameter Mass Cytometry Evaluation of Relapsed/Refractory Multiple Myeloma Patients Treated with Daratumumab Demonstrates Immune Modulation as a Novel Mechanism of Action. Cytom. Part A.

[41] Van de Donk N.W. (2018). Immunomodulatory effects of CD38-targeting antibodies. Immunol. Lett..

[42] Van de Donk N.W., Usmani S.Z. (2018). CD38 Antibodies in Multiple Myeloma: Mechanisms of Action and Modes of Resistance. Front. Immunol..

[43] Campbell K.S., Cohen A.D., Pazina T. (2018). Mechanisms of NK Cell Activation and Clinical Activity of the Therapeutic SLAMF7 Antibody, Elotuzumab in Multiple Myeloma. Front. Immunol..

[44] Dimopoulos M.A., Lonial S., Betts K.A., Chen C., Zichlin M.L., Brun A., Signorovitch J.E., Makenbaeva D., Mekan S., Sy O. (2018). Elotuzumab plus lenalidomide and dexamethasone in relapsed/refractory multiple myeloma: Extended 4-year follow-up and analysis of relative progression-free survival from the randomized ELOQUENT-2 trial. Cancer.

[45] Dimopoulos M.A., Dytfeld D., Grosicki S., Moreau P., Takezako N., Hori M., Leleu X., Leblanc R., Suzuki K., Raab M.S. (2018). Elotuzumab plus Pomalidomide and Dexamethasone for Multiple Myeloma. N. Engl. J. Med..

[46] Trudel S., Moreau P., Touzeau C. (2019). Update on elotuzumab for the treatment of relapsed/refractory multiple myeloma: Patients’ selection and perspective. OncoTargets Ther..

[47] Ochoa M.C., Perez-Ruiz E., Minute L., Oñate C., Perez G., Rodriguez I., Zabaleta A., Alignani D., Fernandez-Sendin M., Lopez A. (2019). Daratumumab in combination with urelumab to potentiate anti-myeloma activity in lymphocyte-deficient mice reconstituted with human NK cells. OncoImmunology.

[48] Guillerey C., Nakamura K., Pichler A.C., Barkauskas D., Krumeich S., Stannard K., Miles K., Harjunpää H., Yu Y., Casey M. (2019). Chemotherapy followed by anti-CD137 mAb immunotherapy improves disease control in a mouse myeloma model. JCI Insight.

[49] Jelinek T., Paiva B., Hajek R. (2018). Update on PD-1/PD-L1 Inhibitors in Multiple Myeloma. Front. Immunol..

[50] Paul B., Kang S., Zheng Z., Kang Y. (2018). The challenges of checkpoint inhibition in the treatment of multiple myeloma. Cell. Immunol..

[51] Sponaas A.M., Waage A., Vandsemb E.N., Misund K., Børset M., Sundan A., Slørdahl T.S., Standal T. (2021). Bystander Memory T Cells and IMiD/Checkpoint Therapy in Multiple Myeloma: A Dangerous Tango?. Front. Immunol..

[52] He Y., Rivard C.J., Rozeboom L., Yu H., Ellison K., Kowalewski A., Zhou C., Hirsch F.R. (2016). Lymphocyte-activation gene-3, an important immune checkpoint in cancer. Cancer Sci..

[53] Yu X., Huang X., Chen X., Liu J., Wu C., Pu Q., Wang Y., Kang X., Zhou L. (2019). Characterization of a novel anti-human lymphocyte activation gene 3 (LAG-3) antibody for cancer immunotherapy. mAbs.

[54] Madduri D., Rosko A., Brayer J., Zonder J., Bensinger W.I., Li J., Xu L., Adriaens L., Chokshi D., Zhang W. (2020). REGN5458, a BCMA x CD3 Bispecific Monoclonal Antibody, Induces Deep and Durable Responses in Patients with Relapsed/Refractory Multiple Myeloma (RRMM). Blood.

[55] Shah N., Chari A., Scott E., Mezzi K., Usmani S.Z. (2020). B-cell maturation antigen (BCMA) in multiple myeloma: Rationale for targeting and current therapeutic approaches. Leukemia.

[56] Topp M.S., Duell J., Zugmaier G., Attal M., Moreau P., Langer C., Kroenke J., Facon T., Einsele H., Munzert G. (2018). Treatment with AMG 420, an Anti-B-Cell Maturation Antigen (BCMA) Bispecific T-Cell Engager (BiTE®) Antibody Construct, Induces Minimal Residual Disease (MRD) Negative Complete Responses in Relapsed and/or Refractory (R/R) Multiple Myeloma (MM) Patients: Results of a First-in-Human (FIH) Phase I Dose Escalation Study. Blood.

[57] Topp M.S., Duell J., Zugmaier G., Attal M., Moreau P., Langer C., Krönke J., Facon T., Salnikov A.V., Lesley R. (2020). Anti–B-Cell Maturation Antigen BiTE Molecule AMG 420 Induces Responses in Multiple Myeloma. J. Clin. Oncol..

[58] Gavriatopoulou M., Ntanasis-Stathopoulos I., Dimopoulos M.A., Terpos E. (2019). Anti-BCMA antibodies in the future management of multiple myeloma. Expert Rev. Anticancer Ther..

[59] Thomas R., Al-Khadairi G., Roelands J., Hendrickx W., Dermime S., Bedognetti D., Decock J. (2018). NY-ESO-1 Based Immunotherapy of Cancer: Current Perspectives. Front. Immunol..

[60] Feng D., Sun J. (2020). Overview of anti-BCMA CAR-T immunotherapy for multiple myeloma and relapsed/refractory multiple myeloma. Scand J. Immunol..

[61] Brudno J.N., Maric I., Hartman S.D., Rose J.J., Wang M., Lam N., Stetler-Stevenson M., Salem D., Yuan C., Pavletic S. (2018). T Cells Genetically Modified to Express an Anti-B-Cell Maturation Antigen Chimeric Antigen Receptor Cause Remissions of Poor-Prognosis Relapsed Multiple Myeloma. J. Clin. Oncol..

[62] Rodríguez-Lobato L.G., Ganzetti M., Fernández de Larrea C., Hudecek M., Einsele H., Danhof S. (2020). CAR T-Cells in Multiple Myeloma: State of the Art and Future Directions. Front Oncol..

[63] Bu D.-X., Singh R., Choi E.E., Ruella M., Nunez-Cruz S., Mansfield K.G., Bennett P., Barton N., Wu Q., Zhang J. (2018). Pre-clinical validation of B cell maturation antigen (BCMA) as a target for T cell immunotherapy of multiple myeloma. Oncotarget.

[64] Raje N., Berdeja J., Lin Y., Siegel D., Jagannath S., Madduri D., Liedtke M., Rosenblatt J., Maus M.V., Turka A. (2019). Anti-BCMA CAR T-Cell Therapy bb2121 in Relapsed or Refractory Multiple Myeloma. N. Engl. J. Med..

[65] Pont M.J., Hill T., Cole G.O., Abbott J.J., Kelliher J., Salter A.I., Hudecek M., Comstock M.L., Rajan A., Patel B.K.R. (2019). γ-Secretase inhibition increases efficacy of BCMA-specific chimeric antigen receptor T cells in multiple myeloma. Blood.

[66] Fan F., Zhao W., Liu J., He A., Chen Y., Cao X., Yang N., Wang B., Zhang P., Zhang Y. Durable remissions with BCMA-specific chimeric antigen receptor (CAR)-modified T cells in patients with refractory/relapsed multiple myeloma. Abstract LBA3001. Proceedings of the 2017 ASCO Annual Meeting.

[67] Smith E.L., Harrington K., Staehr M., Masakayan R., Jones J., Long T.J., Ng K.Y., Ghoddusi M., Purdon T.J., Wang X. (2019). GPRC5D is a target for the immunotherapy of multiple myeloma with rationally designed CAR T cells. Sci. Transl. Med..

[68] Brooks W.H., Netsky M.G., Normansell D.E., Horwitz D.A. (1972). Depressed cell-mediated immunity in patients with primary intracranial tumors. Characterization of a humoral immunosuppressive factor. J. Exp. Med..

[69] Kuppner M.C., Hamou M.F., Sawamura Y., Bodmer S., de Tribolet N. (1989). Inhibition of lymphocyte function by glioblastoma-derived transforming growth factor beta 2. J. Neurosurg..

[70] Uckun F.M., Qazi S., Hwang L., Trieu V.N. (2019). Recurrent or Refractory High-Grade Gliomas Treated by Convection-Enhanced Delivery of a TGFβ2-Targeting RNA Therapeutic: A Post-Hoc Analysis with Long-Term Follow-Up. Cancers.

[71] Ganesh K., Massagué J. (2018). TGF-β Inhibition and Immunotherapy: Checkmate. Immunity.

[72] Mariathasan S., Turley S.J., Nickles D., Castiglioni A., Yuen K., Wang Y., Kadel E.E., Koeppen H., Astarita J.L., Cubas R. (2018). TGFβ attenuates tumour response to PD-L1 blockade by contributing to exclusion of T cells. Nature.

[73] Thomas D.A., Massagué J. (2005). TGF-β directly targets cytotoxic T cell functions during tumor evasion of immune surveillance. Cancer Cell.

[74] Tauriello D.V., Palomo-Ponce S., Stork D., Berenguer-Llergo A., Badia-Ramentol J., Iglesias M., Sevillano M., Ibiza S., Cañellas A., Hernando-Momblona X. (2018). TGFbeta drives immune evasion in genetically reconstituted colon cancer metastasis. Nature.

[75] Teramachi J., Tenshin H., Hiasa M., Oda A., Bat-Erdene A., Harada T., Nakamura S., Ashtar M., Shimizu S., Iwasa M. (2020). TAK1 is a pivotal therapeutic target for tumor progression and bone destruction in myeloma. Haematologica.

[76] Uckun F.M., Carlson J., Orhan C., Powell J., Pizzimenti N.M., van Wyk H., Ozercan I.H., Volk M., Sahin K. (2020). Rejuveinix Shows a Favorable Clinical Safety Profile in Human Subjects and Exhibits Potent Preclinical Protective Activity in the Lipopolysaccharide-Galactosamine Mouse Model of Acute Respiratory Distress Syndrome and Multi-Organ Failure. Front. Pharmacol..

[77] Trieu V., Saund S., Rahate P.V., Barge V.B., Nalk S., Windlass H., Uckun F.M. (2021). Targeting TGF-β pathway with COVID-19 Drug Candidate ARTIVeda/PulmoHeal Accelerates Recovery from Mild-Moderate COVID-19. MedRxiv.

[78] Uckun F.M., Saund S., Windlass H., Trieu V. (2021). Repurposing Anti-Malaria Phytomedicine Artemisinin as a COVID-19 Drug. Front. Pharmacol..

[79] Wang Q., Lu Y., Li R., Jiang Y., Zheng Y., Qian J., Bi E., Zheng C., Hou J., Wang S. (2018). Therapeutic effects of CSF1R-blocking antibodies in multiple myeloma. Leukemia.

[80] Wang S., Ma Y., Wang X., Jiang J., Zhang C., Wang X., Jiang Y., Huang H., Hong L. (2019). IL-17A Increases Multiple Myeloma Cell Viability by Positively Regulating Syk Expression. Transl. Oncol..

[81] Yan H., Zheng G., Qu J., Liu Y., Huang X., Zhang E., Cai Z. (2019). Identification of key candidate genes and pathways in multiple myeloma by integrated bioinformatics analysis. J. Cell. Physiol..

[82] Frenquelli M., Caridi N., Antonini E., Storti F., Viganò V., Gaviraghi M., Occhionorelli M., Bianchessi S., Bongiovanni L., Spinelli A. (2019). The WNT receptor ROR2 drives the interaction of multiple myeloma cells with the microenvironment through AKT activation. Leukemia.

[83] Lorenz J., Waldschmidt J., Wider D., Follo M., Ihorst G., Chatterjee M., May A.M., Duyster J., Rosenwald A., Wäsch R. (2015). From CLL to Multiple Myeloma—Spleen Tyrosine Kinase (SYK) influences multiple myeloma cell survival and migration. Br. J. Haematol..

[84] Koerber R.-M., Held S.A.E., Heine A., Kotthoff P., Daecke S.N., Bringmann A., Brossart P. (2015). Analysis of the anti-proliferative and the pro-apoptotic efficacy of Syk inhibition in multiple myeloma. Exp. Hematol. Oncol..

[85] Uckun F.M., Qazi S., Cely I., Sahin K., Shahidzadeh A., Ozercan I., Yin Q., Gaynon P., Termuhlen A., Cheng J. (2013). Nanoscale liposomal formulation of a SYK P-site inhibitor against B-precursor leukemia. Blood.

[86] Asimakopoulos F., Kim J., Denu R.A., Hope C., Jensen J.L., Ollar S.J., Hebron E., Flanagan C., Callander N., Hematti P. (2013). Macrophages in multiple myeloma: Emerging concepts and therapeutic implications. Leuk. Lymphoma.

[87] Van Dalen F.J., van Stevendaal M.H.M.E., Fennemann F.L., Verdoes M., Ilina O. (2019). Molecular repolarisation of tumour-associated macrophages. Molecules.

[88] Tamura H., Ishibashi M., Sunakawa M., Inokuchi K. (2019). Immunotherapy for Multiple Myeloma. Cancers.

[89] Lu C., Meng S., Jin Y., Zhang W., Li Z., Wang F., Wang-Johanning F., Wei Y., Liu H., Tu H. (2017). A novel multi-epitope vaccine from MMSA-1 and DKK1 for multiple myeloma immunotherapy. Br. J. Haematol..

[90] Packiriswamy N., Upreti D., Zhou Y., Khan R., Miller A., Diaz R.M., Rooney C.M., Dispenzieri A., Peng K.-W., Russell S.J. (2020). Oncolytic measles virus therapy enhances tumor antigen-specific T-cell responses in patients with multiple myeloma. Leukemia.

[91] Wu S., Kuang H., Ke J., Pi M., Yang D.-H. (2021). Metabolic Reprogramming Induces Immune Cell Dysfunction in the Tumor Microenvironment of Multiple Myeloma. Front. Oncol..

[92] Brooks G.A. (2018). The Science and Translation of Lactate Shuttle Theory. Cell Metab..

[93] Li F., Simon M.C. (2020). Cancer Cells Don’t Live Alone: Metabolic Communication within Tumor Microenvironments. Dev. Cell.

[94] Doherty J.R., Cleveland J.L. (2013). Targeting lactate metabolism for cancer therapeutics. J. Clin. Investig..

[95] Borsi E., Perrone G., Terragna C., Martello M., Dico A.F., Solaini G., Baracca A., Sgarbi G., Pasquinelli G., Valente S. (2014). Hypoxia inducible factor-1 alpha as a therapeutic target in multiple myeloma. Oncotarget.

[96] Nagao A., Kobayashi M., Koyasu S., Chow C.C.T., Harada H. (2019). HIF-1-Dependent Reprogramming of Glucose Metabolic Pathway of Cancer Cells and Its Therapeutic Significance. Int. J. Mol. Sci..

[97] Cohen A.D., Garfall A.L., Stadtmauer E.A., Melenhorst J.J., Lacey S.F., Lancaster E., Vogl D.T., Weiss B.M., Dengel K., Nelson A. (2019). B cell maturation antigen-specific CAR T cells are clinically active in multiple myeloma. J. Clin. Investig..

[98] Suurs F.V., Hooge M.N.L.-D., de Vries E.G., de Groot D.J.A. (2019). A review of bispecific antibodies and antibody constructs in oncology and clinical challenges. Pharmacol. Ther..

[99] Mateos M.-V., Cavo M., Blade J., Dimopoulos M.A., Suzuki K., Jakubowiak A., Knop S., Doyen C., Lucio P., Nagy Z. (2020). Overall survival with daratumumab, bortezomib, melphalan, and prednisone in newly diagnosed multiple myeloma (ALCYONE): A randomised, open-label, phase 3 trial. Lancet.

[100] Dimopoulos M.A., Moreau P., Terpos E., Mateos M.-V., Zweegman S., Cook G., Delforge M., Hájek R., Schjesvold F., Cavo M. (2021). Multiple Myeloma: EHA-ESMO Clinical Practice Guidelines for Diagnosis, Treatment and Follow-up. Hemasphere.

[101] Moreau P., Mateos M.-V., Berenson J.R., Weisel K., Lazzaro A., Song K., Dimopoulos M.A., Huang M., Zahlten-Kumeli A., Stewart A.K. (2018). Once weekly versus twice weekly carfilzomib dosing in patients with relapsed and refractory multiple myeloma (A.R.R.O.W.): Interim analysis results of a randomised, phase 3 study. Lancet Oncol..

[102] Shah J., Usmani S., Stadtmauer E.A., Rifkin R.M., Berenson J.R., Berdeja J.G., Lyons R.M., Klippel Z., Chang Y.-L., Niesvizky R. (2019). Oprozomib, pomalidomide, and Dexamethasone in Patients with Relapsed and/or Refractory Multiple Myeloma. Clin. Lymphoma Myeloma Leuk..

[103] Kumar S.K., Grzasko N., Delimpasi S., Jedrzejczak W.W., Grosicki S., Kyrtsonis M., Spencer A., Gupta N., Teng Z., Byrne C. (2019). Phase 2 study of all-oral ixazomib, cyclophosphamide and low-dose dexamethasone for relapsed/refractory multiple myeloma. Br. J. Haematol..

[104] Chari A., Vogl D.T., Gavriatopoulou M., Nooka A.K., Yee A.J., Huff C.A., Moreau P., Dingli D., Cole C., Lonial S. (2019). Oral Selinexor–Dexamethasone for Triple-Class Refractory Multiple Myeloma. N. Engl. J. Med..

[105] Alfarra H., Weir J., Grieve S., Reiman T. (2020). Targeting NK Cell Inhibitory Receptors for Precision Multiple Myeloma Immunotherapy. Front. Immunol..

[106] Pinto V., Bergantim R., Caires H.R., Seca H., Guimarães J.E., Vasconcelos M.H. (2020). Multiple Myeloma: Available Therapies and Causes of Drug Resistance. Cancers.

[107] Grywalska E., Sosnowska-Pasiarska B., Smok-Kalwat J., Pasiarski M., Niedźwiedzka-Rystwej P., Roliński J. (2020). Paving the Way toward Successful Multiple Myeloma Treatment: Chimeric Antigen Receptor T-Cell Therapy. Cells.

[108] Tognarelli S., Wirsching S., Von Metzler I., Rais B., Jacobs B., Serve H., Bader P., Ullrich E. (2018). Enhancing the Activation and Releasing the Brakes: A Double Hit Strategy to Improve NK Cell Cytotoxicity Against Multiple Myeloma. Front. Immunol..

[109] Lesokhin A.M., Bal S., Badros A.Z. (2019). Lessons Learned from Checkpoint Blockade Targeting PD-1 in Multiple Myeloma. Cancer Immunol. Res..

